# Does social support effect knowledge and diabetes self-management practices in older persons with Type 2 diabetes attending primary care clinics in Cape Town, South Africa?

**DOI:** 10.1371/journal.pone.0230173

**Published:** 2020-03-13

**Authors:** Mahmoud M. Werfalli, Sebastiana Z. Kalula, Kathryn Manning, Naomi S. Levitt

**Affiliations:** 1 Chronic Disease Initiative for Africa, Cape Town, Western Cape, South Africa; 2 Division of Endocrinology and Diabetic Medicine, Department of Medicine, Faculty of Health Science, University of Cape Town, Cape Town, Western Cape, South Africa; 3 Division of Geriatric Medicine, Department of Medicine, Faculty of Health Sciences, The Albertina and Walter Sisulu Institute of Ageing in Africa, Cape Town, South Africa; University of Wisconsin Madison School of Pharmacy, UNITED STATES

## Abstract

**Background:**

In South Africa with one of the most rapidly ageing populations in Africa despite the demographic impact of the HIV/AIDS epidemic, diabetes is a major cause of morbidity and mortality. Self-management is challenging for all those with the condition but is likely to create a higher demand for those who may have existing co-morbidities associated with age, and long-standing chronic diseases.

**Objective:**

To determine the relationship of social support, especially that of family and friends with their self-management.

**Methods:**

This cross-sectional study was undertaken in the Cape Town metropole primary care clinics. The sample comprised 406 people drawn from four community health centres (CHC) that are served by Groote Schuur Hospital at the tertiary level.

**Results:**

Of the 406 participants, 68.5% were females, 60.5% were living with a family member, and almost half were married. The mean duration of diabetes from diagnosis was eight years. More than half (57.4%) had no or only primary education. Half the participants (50.2%) had poor knowledge level in relation to symptoms and complications of diabetes. Multivariable linear regression showed older age was associated with poor knowledge (*®*: -1.893, 95% CI-3.754; -0.031) and higher income was associated with self-management practice (*®*: 3.434, 95% CI 0.797; 6.070). Most participants received family support to follow aspects of diabetes self-management. The ordinal logistic regression indicated that family support was positively associated with the self-management practice score for following a diabetic meal plan, taking care of feet, physical activity, testing blood sugar and handling participants’ feelings about being diabetic, but not for taking medication.

**Conclusions:**

Consideration needs to be given to developing and testing education programmes that focus on needs of older people with diabetes and emphases the role of family and friends.

## Introduction

Population ageing has been accompanied by a shift in disease profile to non-communicable diseases (NCD), increased levels of disability, and an increasing loss of physical and cognitive functioning. Most high-income countries have accepted the chronological age of 65 years as a definition of 'elderly' or older person, but like many westernized concepts, this does not adapt well to the situation in Africa. As a result of the changing in legislation via the Social Assistance Amendment Act, 6 of 2008, South Africa aligned itself with the United Nations definition of the ‘older persons’ as all persons over the age of 60 years. [1–2] South Africa has one of the most rapidly ageing populations in Africa despite the demographic impact of the HIV/AIDS epidemic. The population aged 60 years and older numbered over 4.5 million in 2016, thereby accounting for just over 8% of the South African population [3]. It is expected that, by 2025, the proportion of the older population will increase by 10.5% to reach 5.23 million [4–5]. The latest estimates are that three-and-a-half million South Africans (about 6% of the population) have diabetes, and there are many more who are undiagnosed. This number is anticipated to grow by 30% by the year 2030. [6–7] According to the WHO Global Report on diabetes in 2016, the combination of increasing prevalence of diabetes and increasing life expectancy in many populations with diabetes may be leading to a shift in the types of morbidity that accompany diabetes, such as cancers and cognitive disability. [8] Inevitably, this will place further strain on both healthcare resources and health providers.

In South Africa, 80% of the population receive their health care through the government funded public sector. [9] In general, community health centres (CHC) and smaller primary care clinics are the older persons’ first point of contact with the healthcare services. These are staffed according to their size and location and provide a comprehensive package of care. The CHCs are overcrowded and poorly resourced due to the multiple disease burden, leaving limited time for the front-line health workers to deal with the management of patients with diseases such as diabetes. [9] A qualitative study found that patients with diabetes in this setting were ill-equipped to play an active role in self-care due to their limited opportunities for education and counselling. [10] In this setting too, poor control of glycaemia and hypertension together with high levels of multimorbidity are commonly encountered. [11]

Diabetes self-management practices (DSMP) form the foundation of diabetes care. These involve knowledge, skills and motivation as it requires, amongst others, adjustment of the diet, monitoring of blood glucose levels, where appropriate and an increase in physical exercise. [12,13] Sprague et al. found that the decreased priority given to patient education among older individuals, their support systems, and the healthcare community is a factor that negatively impacts their learning diabetes self-management. [14]

A number of barriers associated with ageing reduce the older persons’ potential for engaging with traditional self-management education programmes such as lectures/group sessions; for example, hearing and visual deterioration. Further self-management practice may be affected by reduced manual dexterity due to osteoarthritis which is common in this group. [15–16] Therefore, older people might have fewer resources available to manage their condition than younger people and will then have a higher need for self-management support. The loss of friends and family makes them more vulnerable to loneliness and social isolation. [17]

Poor social support is associated not only with an increase in mortality, morbidity and psychological distress but a decrease in overall general health and well-being. [18] Several studies have found social support vital to SMP in people with chronic diseases. [18–19] Social support can be either emotional or physical. Emotional, social support is defined as the degree to which interpersonal relationships serve the purpose of providing emotional, informational or influential quality of life for the individual. [20] Physical support is defined as the forms and numbers of social relationships (marital status, the number of friends) and the degree of connection between these relationships (social network).

Most frequently social support for persons with diabetes covers aspects of active support and emotional encouragement with taking medications, monitoring blood glucose, foot and eye care, following diabetic meal plans and increasing physical activity. [21] This study was undertaken in older people with diabetes in South Africa to examine their knowledge about living with and managing their diabetes; and to determine the relationship of social support, especially that of family and friends with their self-management.

## Methods

### Study design and selection of participants

This cross-sectional study was undertaken in the Cape Town metropole where working class people receive care through a network of primary care clinics. Eighteen community health centers (CHCs) formed the sampling frame for the study based on them being served by Groote Schuur Hospital at the tertiary level. Four CHCs were selected based on population density of the older population in their drainage area as reported in the statistical censuses of 2011 [4].

As a diabetes register system does not exist in the metropole, the population of adults (>60 years) with diabetes within it is not known. Consequently, calculation of a representative sample size was not possible. A purposive convenience sample of (n = 406) was drawn by a random sampling technique from those who attended the four CHCs with approximately (100) participants from each clinic.

The study was approved by the Human Ethics Committee of the University of Cape Town. (HEC REF: 21/2013). This research was conducted in accordance with the Declaration of Helsinki and written consent was obtained from each participant prior to their participation.

### Instruments

We adopted a validated KAP questionnaire from P & T Journal, Medimedia USA, Inc, which was developed by Palaian et al., 2006 [22] and adapted it for this study. The questionnaire has been used in previous KAP studies among diabetics and has proven to be reliable, Cronbach alpha value 0.72. The questionnaire has been used in KAP studies in older adults in low and middle income (LMIC) including South Africa. [22–28]. The questionnaire adapted for this study had close ended and Likert scale questions. The questionnaire contained 7 demographic questions, 14 questions about lifestyle and whether they had received various aspects of care when they attended for routine clinical care in the past year, 11 diabetes specific questions relating to diagnosis and management/medication/monitoring, 11 questions on knowledge of diabetes, 6 questions on tangible and 6 questions on emotional social support (S1 File). We piloted the appropriateness, reliability and clarity of the questionnaire in a sample of 40 participants (10%). The English version questionnaire was translated to Xhosa and Afrikaans, the languages of the study area.

Specifically, knowledge was measured using a 30-item questionnaire. There were 11 multiple choice format questions which assessed some basic knowledge of diabetes ranging from definition of diabetes, major cause, symptoms, complications, monitoring tools, risks of comorbid conditions such as increased blood pressure, a healthy lifestyle, a balanced diet, foot care, the use of medical therapy and eye screening. The respondent would score one point for each correct answer, and some questions had multiple correct options (e.g. symptoms). All points were summed to obtain a total knowledge score ranging from 0–30 from the 11 questions. For ease of interpretation the total score was transformed to give a range from 0 to 100. The Cronbach’s alpha for the knowledge assessment tool was 0.67 (95% Confidence Interval: 0.61 to 0.73).

The Diabetic Self-Management Practice (DSMP) was measured using a 6-item questionnaire with mixed type of response ranging from 0 (no practice) to 4 (more or always practice). The assessment took into account the respondent’s smoking status, physical activity during the past seven days and following a diabetic eating plan. A respondent who practiced all the three: never smoked, did more exercise and followed a diabetic eating plan scored 2 extra points than those who did not. All points were summed to obtain a SMP score for each respondent, which ranged from 0–11. A score ≥6 we regarded as good SMP (Poor score (0–5), Good score (6–11)). Cronbach’s alpha for internal consistency was 0.44 (0.31 to 0.57) for the SMP tool.

We used the Social Support subscale of the Diabetes Care Profile (DCP) [29]. It includes twelve questions related to family and friend social support by adding up the six tangible support variables (Follow meal plan, Take medicine, Take care of feet, Get activity, Test sugar and Handle feelings) and the six emotional support variables (Accept me, Feels uncomfortable, Encourage me, Discourage me, Listen to me, Nag me). The level of social support was assessed using a five-point Likert scale (strongly Disagree, Disagree, Neutral, Agree or Strongly Agree). For the positively worded variable, a score of Agree or Strongly Agree was coded as 1 and that of Strongly Disagree, Disagree, Neutral was coded as 0. For the negatively worded variable, the reverse coding applied. The raw scores ranged from 0 to 12. To simplify interpretation of the scoring, the scores were transformed to a score ranging from 0 to 100, i.e., a score of 12/12 was 100. A higher score meant greater social support. A score >50% that is 7/12 and above was considered as good social support and a score 6/12 or less was considered poor support. The Cronbach’s alpha for physical support was 0.763 and for Emotional support was 0.623.

The afore mentioned transformation of the scores for K, DSMP and SS done for a participant were as follows: K-index = K score / 30 x 100; DSMP-index = DSMP score / 11 * 100; and SS-index = SS score / 12 * 100.

A review of clinic notes to record the HbA1c readings of the last three clinic visits was conducted to assess glycaemic control on patients who had completed the questionnaire. An overall HbA1c level was calculated as the average of the three separate readings. The interval between the readings varied for each participant, dependent on the appointment schedule. Glycaemia control was considered good, acceptable or poor when the HbA1c level was lower than 7.5 between 7.5–8.5% or greater than 8.5%, respectively. [30]

### Data collection

Six fieldworkers were responsible for data collection using questionnaires and a review of medical records for HbA1c and blood glucose results, at the diabetic clinics in the four community health centres. They were trained in the administration of data collection tools, research ethics and an approach to interviewing older persons. Patients with diabetes aged 60 years and over attending the four clinics on the date of data collection were approached for participation in the study. The study was conducted from April to October 2015. Questionnaires were completed for those who agreed to participate. Signed consent to participate in the study was obtained before administration of the study questionnaire. The number of those who refused to participate in the study was not recorded. The fieldworkers were closely supervised by the research team to guarantee the quality of data collection. A random sample of ten questionnaires was checked for completeness and correctness. A total of 413 questionnaires were completed but seven were excluded from the analysis as the participants were below the age of 60 years.

### Data analysis

Data was managed and analysed using SPSS Statistics version 23, [31] and Stata 15.1 (Stata Corp College Station, TX, USA). Categorical data was summarised as frequency and proportions, and continuous data as mean and standard deviation (SD). Unpaired t-tests and one-way analysis of variance (ANOVA) were used to compare knowledge, self-management practice and social support scores between two and three (or more) group variables respectively.

Ordinal logistic regression was used to determine associations between outcomes (knowledge, self-management practice) with components of social support scale. Multivariable linear regression was used to evaluate the associations between outcomes (knowledge, self-management practice) and sociodemographic variables, glycaemia control, and social support (S3 and S4 Tables). Regression estimates were reported with 95% CIs. Statistical significance was indicated by p<0.05.

## Results

### Descriptive and bivariate analysis

#### Patients’ demographic profile and clinical characteristics

Socio-demographic and clinical characteristics of the study sample are presented in Table 1. Of the 406 participants, 68.5% were females, 60.5% were living with a family member, and almost half were married. Two hundred thirty-three (57.4%) had less than 7 years of education. Unsurprisingly most of the participants were pensioners 374 (92%) and 348 (84%) reported family income ≤R 1500 (US$107) per month.

**Table 1:** Socio-demographic and clinical characteristics of the study participants.

			Age group	Gender
Variable	N	(%)	P-value	P-value
Socio-demographic characteristics				
*Age group (Years)*				
60–69	257	63.5		
70–79	121	29.8		
80 or above	28	6.7		
*Sex*: Female	278	68.5		
*Marital status*			0.000	0.000
Single	44	10.7		
Married	209	51.9		
Divorced	38	9.2		
Separated	27	6.5		
Co-habiting	88	21.7		
*Level of Education*			0.35	0.002
≤ 7 years	233	57.4		
9–12 years	162	39.9		
≥ 13 years	11	2.7		
*Employment status*			0.315	0.109
Pensioner: Yes	374	92.0		
*Who are you living with*			0.000	0.000
Spouse	52	12.8		
Family member	246	60.5		
Friend	9	2.2		
Alone	25	6.3		
More than one	74	18.2		
Monthly family income			0.113	0.20
≤R1 500	348	85.7		
>R1 500	58	14.3		
Clinical characteristics				
*Diabetes duration*			0.050	0.28
Less than 5 years	125	31.0		
5–10 years	158	39.2		
>10 years	120	29.7		
Taking prescribed medication: Yes	403	99.3	0.90	0.24
*Type of prescribed medication*			0.39	0.80
Insulin injections	28	6.9		
Pills	250	62.0		
Both	125	31.0		
*Have you experienced low blood sugar*: Yes	73	18.0	0.31	0.016
*Have you experienced high blood sugar*: Yes	195	48.0	0.088	0.004
*Receiving medication for hypertension*: Yes	306	75.4	0.46	0.006
*Receiving medication for heart disease*: Yes	61	15.0	0.33	0.78
*Receiving medication for other chronic disease(s)*: Yes	145	35.7	0.21	0.27
*Blood glucose level*			0.27	0.18
< 90 mg/dl (<5.0 mmol/l)	1	0.2		
90–130 mg/dl (5.0–7.2 mmol/l)	71	17.7		
> 130 mg/dl (>7.2 mmol/l)	330	82.1		
*Glycaemia level (HBA1c)*			0.45	0.22
<7.5%	112	28.9		
7.5–8.5%	91	23.5		
>8.5%	185	47.7		

The mean duration of diabetes from diagnosis was eight years. Sixty-two percent were using oral therapy for glycaemic control, 31% combined insulin and oral agents and 7% insulin alone. The majority were taking medication for other conditions: 306 (75.4%) for hypertension, 138 (34%) for other chronic diseases and 61 (15%) for heart problems. The HbA1c was higher than 8.5% in nearly 185 (47.7%) of the participants, <7.5% in 112 (28.9%) and 7.5–8.5% in 91 (23.4%).). Women were more likely than men to self-report experiencing low and high blood sugar (21% vs 11%, p = 0.016) and (53% vs 37%, p = 0.004) respectively.

#### Knowledge, diabetes self-management practices, social support scores

**Fig 1:**
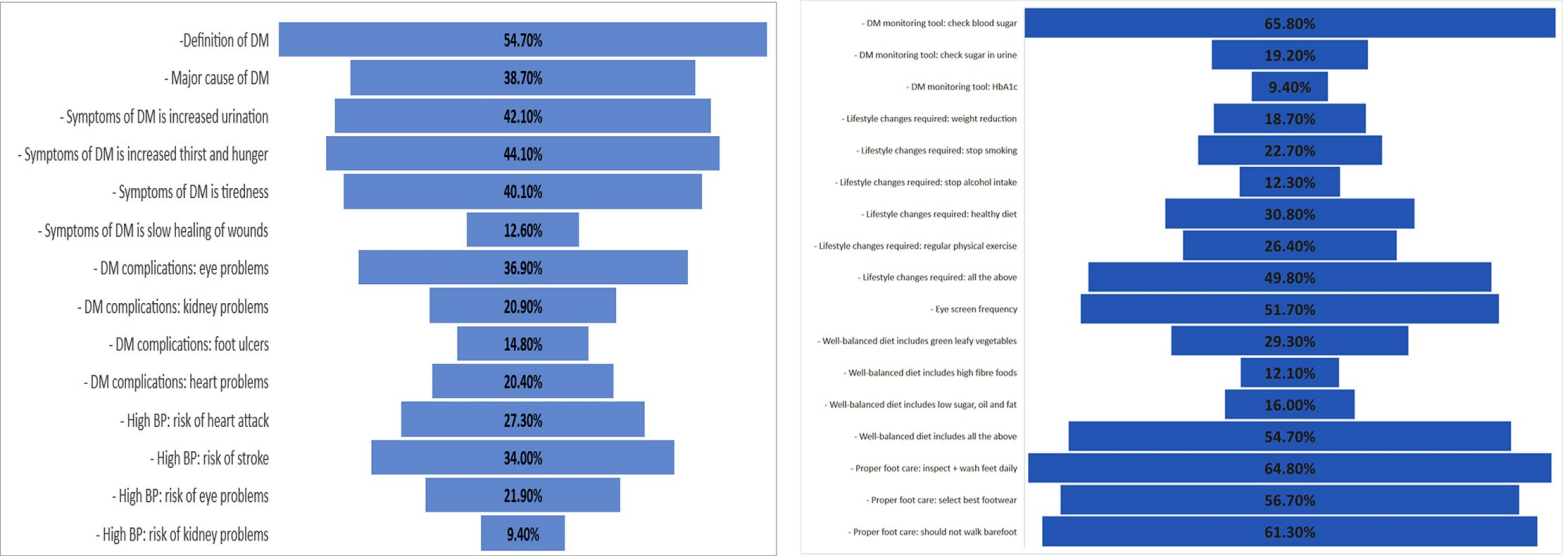
a. Distribution of correct answers to questions on knowledge on symptoms and complications of diabetes and hypertension (n = 406). b Distribution of correct answers to questions on diabetes self-management knowledge (n = 406).

A ‘good knowledge’ score was for those whose score for correct answers was ≥50%. Overall the level of knowledge was poor in 204 (50.2%) (Table 2). The deficiencies were particularly noticeable in relation to symptoms of diabetes and complications of diabetes and hypertension. There was a better level of knowledge about aspects of self-management e.g., a healthy diet and foot care; with 61.3% and 64.8% of respondents correctly answering questions about walking barefoot and daily foot inspection respectively (Fig 1A and 1B).

**Table 2:** Knowledge, diabetes self-management practices, social support scores.

	Score range	N	%
**Knowledge (K)**	Poor K **0–14)**	204	50.2
	Good K **(15–30)**	202	49.8
	Total	406	100.0
**Self-management practice (SMP)**	Poor SMP **(0–5)**	162	39.9
	Good SMP **(6–11)**	244	60.1
	Total	406	100.0
**Social support (SS)**	Poor Social support **(0–6)**	94	23.2
	Good SS **(7–12)**	312	76.8
	Total	406	100.0

Similarly, 233 (57.5%) were assessed as having a good level of physical exercise and almost two-thirds (262 (64.5%)) of the participants were following a diabetic eating plan (Table 3). Over three-quarters of participants (312 (76.8%)) had good social support (Table 2). The majority of participants agreed that their family supported them to follow all aspects of self-care included in the questionnaire and encouraged them in managing their diabetes.

**Table 3:** Self-management practice of lifestyle risk factor.

				Age group	Gender
		N	%	P-value	P-value
**Physical activity in the past week**	Never (0 days)	20	4.9	0.49	0.97
	Seldom (1–2 days)	77	19.0		
	Sometimes (3–4 days)	75	18.5		
	Often (5–7 days)	233	57.5		
**Following diabetic eating plan**	Yes	262	64.5	0.51	0.85
	No	144	35.5		
**How often do YOU test your blood glucose**	Occasionally or 1-2x per week	163	40.1	0.64	0.004
	Once a day	60	14.8		
	3-4x per day	29	7.1		
**Smoking status**	Currently smoking	112	27.7	0.007	0.000
	Previously smoked	147	36.3		
	Never smoked	146	36.0		

Only 74 (18.4%) reported that their family nagged them about their diabetes and 71 (18%) reported that their family felt uncomfortable about them because of their diabetes (Table 4). The mean diabetes knowledge, self-management practice and social support scores of the participants by socio-demographic and clinical characteristics are presented in S1 and S2 Tables. The mean knowledge score was significantly lower for a single (41.2 (SD = 2.9)) compared to married respondents (46.7 (SD = 12.5)) or those in a companionship (49.4 (SD = 12.7)). The mean knowledge score was greater for participants with higher education level and those who had experienced high or low blood sugar (p = 0.005 and p = 0.001 respectively), and those receiving medications for chronic diseases other than hypertension (p = 0.038) than their counterparts.

**Table 4:** Social support assessment of the study participants (n = 406).

(My family or friends help and support me a lot to):	Strongly Disagree (N) %	Somewhat Disagree (N) %	Neutral (N) %	Somewhat Agree (N) %	Strongly Agree (N) %
**a. Follow my meal plan.**	(40) 10.0	(11) 2.7	(27) 6.7	(42) 10.4	(279) 69.4
**b. Take my medicine.**	(43) 10.6	(10) 2.5	(33) 8.1	(30) 7.4	(275) 67.7
**c. Take care of my feet.**	(61) 15.3	(20) 5.0	(34) 8.5	(45) 11.3	(226) 56.5
**d. Get enough physical activity.**	(71) 17.7	(25) 6.2	(24) 6.0	(68) 17.0	(196) 48.9
**e. Test my sugar.**	(95) 24.4	(9) 2.3	(40) 10.3	(24) 62	(175) 45.0
**f. Handle my feelings about diabetes.**	(35) 8.7	(11) 2.3	(22) 10.3	(66) 16.4	(264) 65.7
**My family or friends:**					
**a. Accept me and my diabetes.**	(1) 0.2	(1) 0.2	(2) 0.5	(4) 1.0	(392) 65.7
**b. feels uncomfortable about me because of my diabetes.**	(296) 73.4	(13)3.2	(8) 2.0	(13) 3.2	(71) 17.6
**c. Encourage or reassure me about my diabetes.**	(22) 5.5	(4)1.0	(19) 4.7	(33) 8.2	(320) 79.4
**d. Discourage or upset me about my diabetes.**	(342) 84.9	(18) 4.5	(5) 1.2	(20) 5.0	(14) 3.5
**e. Listen to me when I want to talk about my diabetes.**	(23) 5.7	(7) 1.7	(38) 9.4	(30) 7.4	(293) 72.7
**f. Nag me about diabetes.**	(286) 71.0	(20) 5.0	(21) 5.2	(33) 8.2	(41) 10.2

The social support score means also differed significantly with living arrangements; it was lowest in those living with a spouse (67.7 SD = 21.0), intermediate in those living alone (75.0 SD = 21.5) and highest in those living in multiple household members (82.3 SD = 18.0 p = 0.001). On the other hand, there were no significant associations between diabetic knowledge, self-management practice and social support score with duration of diabetes, the type of medication used to treat diabetes, or receiving treatment for hypertension.

#### The multivariable regression models

The ordinal logistic regression models of knowledge, self-management practice and the components of social support scale are given in Table 5. The table shows the effect of the K and SMP indices on the 12 individual SS components, which were measured on a Likert scale. Social support was positively associated with the self-management practice score for following a diabetic meal plan, taking care of feet, physical activity, handling participants’ feelings about being diabetic and testing blood sugar, but not for taking medication. Family and/or friend emotional support (nagging, encouraging /reassuring and handling feelings about being diabetic) were positively associated with knowledge score. Multivariable linear regression analysis (S3 and S4 Tables) showed older age was negatively associated with knowledge (*®*: -1.893, 95% CI-3.754; -0.031) and higher income was positively associated with self-management practice (*®*: 3.434, 95% CI 0.797; 6.070). There were no significant associations between socio-demographic variables, HbA1c and social support with knowledge or self-management practice.

**Table 5:** The ordinal logistic regression models of knowledge, self-management practice with the components of social support scale.

	Knowledge Index					Self-Management Practices Index					
Parameter Estimates				95% Confidence Interval					95% Confidence Interval		
	Estimate	Std. Error	Sig.	Lower Bound	Upper Bound	Estimate	Std. Error	Sig.	Lower Bound	Upper Bound	Pseudo R2
**My family or friends help and support me a lot to:**											
**a. Follow my meal plan.**	.000	.008	.972	-.016	.015	.024	.006	**.000**	.012	.037	0.023–0.053
**b. Take my medicine.**	.000	.008	.970	-.015	.016	.006	.006	.273	-.005	.018	0.001–0.003
**c. Take care of my feet.**	-.004	.007	.563	-.017	.009	.018	.005	**.000**	.008	.028	0.014–0.037
**d. Get enough physical activity.**	-.002	.006	.769	-.014	.010	.017	.005	**.000**	.008	.027	0.013–0.035
**e. Test my sugar.**	-.011	.007	.114	-.024	.003	.012	.005	**.017**	.002	.021	0.009–0.024
**f. Handle my feelings about diabetes.**	.015	.007	**.042**	.001	.029	.021	.006	**.000**	.010	.032	0.022–0.051
**My family or friends:**											
**a. Accept me and my diabetes.**	-.164	.298	.582	-.749	.421	.250	.212	.240	-.167	.666	0.004–0.019
**b. feels uncomfortable about me because of my diabetes.**	-.003	.009	.721	-.022	.015	.001	.007	.860	-.012	.015	0.00–0.001
**c. Encourage or reassure me about my diabetes.**	.033	.012	**.006**	.009	.056	.013	.009	.166	-.005	.031	0.016–0.030
**d. Discourage or upset me about my diabetes.**	-.020	.019	.301	-.057	.018	.001	.014	.925	-.027	.030	0.002–0.004
**e. Listen to me when I want to talk about my diabetes.**	.010	.009	.295	-.009	.028	.004	.007	.548	-.010	.018	0.002–0.005
**f. Nag me about diabetes.**	.023	.009	**.013**	.005	.040	-.001	.006	.829	-.013	.010	0.009–0.020

## Discussion

In this study, half of the participants had poor knowledge about diabetes and its complications. Just under two-thirds were assessed as having a good level of physical exercise and two-third of the participants were following a diabetic eating plan. Three quarters perceived that their family supported them to follow all aspects of self-care management. Being in the high-income group was associated with good level of self-management practice. Finally, social support was positively associated with both knowledge and a number of self-care aspects. The deficiencies noted in the participant's knowledge relating to the complications of diabetes and hypertension are alarming.

Education of patients including those with diabetes is essential for self-management. It suggests in this study that whatever diabetes educational opportunities participants, particularly the older group, have been exposed to, have not been effective. There are many potential reasons for this. For instance, the high patient numbers and multiple disease burden in primary care clinics, are likely to negatively impact on the time available for patient education by health promoters, nurses or doctors [32]. Other factors to be taken into consideration include lack of attendance at educational sessions when they take place, communication barriers, such as poor hearing, lack of concentration, inability to engage with the material presented and use of didactic modes of communication [33]. However, the participants seemed to have a better knowledge of self-management practice such as foot care and healthy eating. Whether this is because these messages are practical and easier to convey or that the information comes from multiple sources and not only health care workers is uncertain.

To date, there is a scarcity of evidence regarding diabetes self-management education and support in older adults [34]. Some studies that included older persons suggest that this group needs diabetes self-management education that stresses problem-solving skills rather than “rules” to follow [35]. For example, The Diabetes Education and Self-Management Ongoing and Newly Diagnosed (DESMOND) educators observed that older persons contributed to the group and brought valuable experience, but that they may have required a different approach at times [36]. However, no specific examples of such approaches were given in their study.

Sinclair et al. reported that older people benefitted more than middle-aged people from a highly structured group diabetes self-management education intervention with embedded cognitive behavioural strategies compared to standard group education or individual sessions with dietitians and nurse educators [37]. As older persons may have difficulties concentrating and understanding abstract concepts, there is a need for educational material to be provided in the form of simple messages, delivered in a style that engages the person with diabetes and is personalized to their needs, with the emphasis on what they need to know, rather than all there is to know about diabetes [38,39]. Notably, these concepts to enhance knowledge and self-management practices are not unique to the older person with diabetes and are relevant in all societies. Income and financial issues are possible barriers to optimum self-management for many older diabetic patients because of the costs of blood glucose testing, medication and following diet recommendations [39].

Management of chronic disease require support from a patient’s network. We found that almost 75% of participants perceived that their family supported them to follow all aspects of self-care management. Earlier studies have shown that social support and social networks influence health behaviours and health outcomes [40–41]. For example, a study of family behaviours and relationships to adherence and metabolic control, individuals with diabetes negative perceptions of support from family were associated with lower adherence to diabetes management areas (i.e. glucose testing, diet adherence, and insulin injections). On the other hand, positive impacts have been shown to affect an individual’s management of the disease. For instance, Sinclair et al. found that adherence to self-care regimens (i.e. insulin treatments, monitoring blood glucose, exercise, and self-care away from home) was associated with emotional and instrumental support from friends and family [42]. This suggests that the perceived availability and knowledge of friends and family as being present positively impacts self-management efforts of individuals with diabetes.

Furthermore, Connell et al found that social support had only a positive association with general morale among women, while there was a direct correlation between social support and adherence to treatment among older men with diabetes [43]. Among older persons, it has been found that women tend to exhibit better self-care behaviour, are less likely to be married, and are more likely to discuss personal issues with friends than men are [43]. This is in line with our study that showed that women tested their glucose levels more frequently than men.

In contrast, men are more likely to have a family member who assists with various aspects of their self-care regimen. The self-care behaviour of older women with diabetes is also influenced by social role obligations, and this is especially true of certain communities like the South African community, where women often bear a bigger responsibility as the caregiver for the whole family [43]. Unsurprisingly, such women also report a lower quality of life as well as encountering more barriers to the self-management of their diabetes [43]. Weaver et.al reported that both symptoms of diabetes and difficulties achieving socially important roles contribute to poor mental health among Indian diabetic women.[44]

Diabetes self-management education (DSME) is an integral part of diabetes care “for all people with diabetes who want to attain successful health-related outcomes,” irrespective of age [44]. It is important to be aware of current DSME guidelines for older persons and how these guidelines can be implemented in a clinical setting. However, older persons are under-represented in DSME research studies, so evidence-based guidelines specifically targeted toward older individuals are challenging to formulate [45].

The American Association of Diabetes Educators (AADE) and the American Geriatric Society (AGS) have formulated guidelines for DSME in the older adults mainly based on expert consensus [46,47]. For example, Older persons who are experiencing difficulties with daily tasks such as hearing loss, vision problems, decreased mobility and falls, will need individual rather than group DSME. Indeed, learning new skills will take longer and may require referral to a visiting nurse/CHW to make sure the task is fully integrated into the person's self-care regimen. If needed, family members or other caregivers should be included in DSME [47].

Additional information about the influence of social support on chronic illness self-management has been supported by research. A systematic review reported evidence for a modest positive relationship between social support and chronic illness self-management, particularly for diabetes [48]. The finding was that a large information network is beneficial for self-management capabilities, especially in low education populations. This may be of an advantage in many cultures such as in South Africa where strong family relationships and family caring are important and highly valued [49–51].

A cohesive and supportive family may provide older diabetic patients with an opportunity to express feelings and fears. When DSMP is reviewed as a shared responsibility with the whole family, older persons may adopt DSMP activities more easily and feel more self-confident in managing diabetes [52]. As family-focused interventions may be more effective in improving DSMP performance than individual-focused interventions, including family members or friends in education programmes should be considered [53–56].

However, the shortage of professional health care workers in South Africa highlights the need to develop alternative delivery models for education and self-management for people with diabetes who attend primary care services. These include using the services of community health workers (CHWs) and peer supporters and should draw on previous lessons learnt [56]. For example, while a pragmatic trial of a group diabetes education programme led by health promoters in Cape Town improved blood pressure, but not self‐efficacy, locus of control or glycaemia control; process evaluation suggested numerous problems. These included, finding suitable space for group education, with patient attendance and with full adoption of a guiding style by the health promoters. Thus, groups held outside of primary care clinics in the community and led by well-trained CHWs or peers may be a better option, so too may the active participation by family members in these groups [57]. In addition, the emphasis on diabetes prevention programs in middle-aged people must be highlighted, because it will enable the next generation of older persons to live with a reduced diabetes burden. For these reasons, South Africa's health care system needs to transform its services offered to older persons to reduce health care costs and ensure quality of life [57].

The various initiatives currently underway to re-engineer the primary healthcare system in SA to more effectively deal with NCDs, will go some way to meeting the identified needs of older diabetic patients and to addressing their barriers to care [58–60].However, as part of this re-modelling exercise, it is perhaps opportune for the health department to consult older chronic care patients and involve them in decision making and the planning of services. This study alerts policymakers and clinicians to some of the specific issues considered to be pertinent and important in the care and management of older persons with diabetes.

As our results show that weak social support is a predictor of both poor knowledge and poor SMP, consideration should be given to health practitioners assessing social support when people with diabetes are reviewed clinically. However, interventions need to be put in place to enhance the level of support. This could include recommending that the person be open to accepting support from family and friends, suggest that the main carer attend some clinic visits with the person or referral of the person to a support group.

### Study strengths and limitation

This study contributes to an understanding and fills a gap in the current knowledge, relating to diabetes self-management practices, and perceived social support from family and friends and diabetes care for older people in South Africa. However, the study has some limitations. First, as a cross-sectional survey design, our study could not assess cause and effect. Second, the measurements of self-report rather than direct observation of self-care practices are recognised as a limitation. In addition, the use of a convenience sample drawn from a population who attend a diabetes clinic excludes those who did not attend. Third, younger participants age <60 years were not included in this study to enable comparison of outcome measures to older participants. Fourth, our study was limited to one region and may not be representative of all older South Africans with diabetes. Lastly, as an assessment tool, we have used a diabetes-related social support scale which we believe was a more specific tool in identifying diabetes specific social support than a more general social support scale. However, it would have been strengthened by adding an open-ended component following the social support scale, (for example asking the participant to list the top 3 ways that family and friends help in managing diabetes, and the 3 ways they help least in diabetes management). This might have provided better insight into what role family and friends play in the management process and inform more appropriate measures and/or items on social support scales.

Future research should focus on developing and evaluating family/friends focused community-based multi-disciplinary education programmes to improve DSMP among older individuals attending primary care clinics with a view to enhancing the quality of life and to reduce disability.

## Conclusions

Consideration needs to be given to developing and evaluating education programmes that focus on the needs of older people with diabetes mellitus and emphases the role of family and friends. However, it is imperative to introduce programmes at a younger age so that diabetes self-management strategies are embedded as a life course perspective to enhance positive outcomes for persons living with diabetes.

## Supporting information

S1 File (DOC)

S1 Table Mean diabetes knowledge, self-management practice and social support scores of the participants by socio-demographic characteristics. (DOCX)

S2 Table Mean diabetes knowledge, self-management practice and social support scores by clinical characteristics of the participants. (DOCX)

S3 Table Association of socio-demographic variables, HbA1c and social support with knowledge score. (DOCX)

S4 Table Association of socio-demographic variables, HbA1c and social support with self-management practice score. (DOCX)
