# Supplementary material for: Does social support effect knowledge and diabetes self-management practices in older persons with Type 2 diabetes attending primary care clinics in Cape Town, South Africa?
Source: PLoS One. 2020 Mar 13;15(3):e0230173. doi: 10.1371/journal.pone.0230173 (PMC7069645; doi:10.1371/journal.pone.0230173)
Supplement: S1 File — (DOC) [file pone.0230173.s001.doc]

**
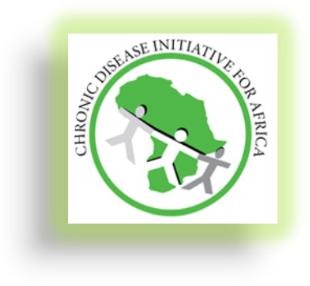

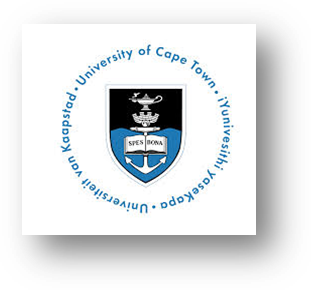
**

**Chronic Disease Initiative in Africa (CDIA) /UCT**

**Title of study: Development of a Self-Management Care Programme for Older people with Type 2 Diabetes attending community health centers in Cape Town Metropole**

**Questionnaire Survey**

**Supervisor: Professor Naomi (Dinky) Levitt**

**Co – Supervisor: Dr Sebastiana Kalula**

**Student: Mahmoud Werfalli**

**Community Health Centre: ……………………………………………………….**

**Interviewer Name: ………………………………………………………………**

**Staff Nr: ………………………………………………………………**

**Date of Interview: ………………………………………………………**

| **GENERAL AND DEMOGRAPHIC INFORMATION** |
| --- |

| **STUDY NUMBER**…………………  **INTERVIEWER’S NAME**  **CLINIC**  **NAME OF SUBJECT**  **ADDRESS OF SUBJECT**    **TELEPHONE no:**  **FOLDER no**:  **DATE OF INTERVIEW :**  **D D M M Y Y Y Y**  ***A) I would like you to give me information about yourself.***  ***(Please check the answer that best describes you)*** | | | | | ┌───┬───┬───┬───┐  │░ ░│░ ░│░ ░│░░░│  └───┴───┴───┴───┘  ┌───┬───┐  │ ░░│ ░░│  ┌───┼───┼───┤  │░ ░│░ ░│ ░░│  ├───┼───┼───┤  │░ ░│░ ░│ ░░│  └───┼───┼───┤  │ ░░│ ░░│  └───┴───┘  ┌───┬───┐  │░ ░│ ░░│  └───┴───┘ |  | |  | | | | | | | |
| --- | --- | --- | --- | --- | --- | --- | --- | --- | --- | --- | --- | --- | --- | --- | --- |
|  | | | | |  |  | |  | | | | | | | |
| **1) In what year are you born?**  **D D M M Y Y Y Y**  2) Gender   | **Male** | **Female** | | --- | --- |     **3) What is your marital status at present?** | | | | |  |  | |  | | | | | | | |
|  | Single | 1 | | |  |  | |  | | | | | | | |
|  | Married | 2 | | |  |  | |  | | | | | | | |
|  | Divorced | 3 | | |  |  | |  | | | | | | | |
|  | Separated | 4 | | |  |  | |  | | | | | | | |
|  | Other | 5 | | |  |  | |  | | | | | | | |
| **4)** **What is the highest level of education you completed?** | | | | | |  | |  | | |  | | | | |
|  | Never | 1 | | |  |  | |  | | | | | | | |
|  | Under grade 6 | 2 | | |  |  | |  | | | | | | | |
|  | Grade 6 | 3 | | |  |  | |  | | | | | | | |
|  | Grade 9 | 4 | | |  |  | |  |  | | | | | | |
|  | Grade 10 | 5 | | |  |  | |  |  | | | | | | |
|  | Grade 11 | 6 | | |  |  | |  |  | | | | | | |
|  | Grade 12 | 7 | | |  |  | |  |  | | | | | | |
|  | College | 8 | | |  |  | |  |  | | | | | | |
|  | University | 9 | | |  |  | |  |  | | | | | | |
| **5a)** **Are you currently working in a paid job?** | | | | | |  | |  | | |  | | | | |
|  | Yes | 1 | | |  |  | |  |  | | | | | | |
|  | No | 2 | | |  |  | |  |  | | | | | | |
| **5b)** **If No, are you?** | | | | | |  | |  | | |  | | | | |
|  | Unemployed (but physically able to work) | 1 | | |  |  | |  |  | | | | | | |
|  | Unable to work due to health problems | 2 | | |  |  | |  |  | | | | | | |
|  | On government grant (eg. Old age pensioner | 3 | | |  |  | |  |  | | | | | | |
| **6)** **Who are the other people living under the same roof?** | | | | | |  | |  |  | | | | | | |
|  | Spouse | 1 | | |  |  | |  |  | | | | | | |
|  | Family member | 2 | | |  |  | |  |  | | | | | | |
|  | Friend | 3 | | |  |  | |  |  | | | | | | |
|  | Alone | 4 | | |  |  | |  |  | | | | | | |
|  | More than one | 5 | | |  |  | |  |  | | | | | | |
| **7) What is your monthly income?** |  |  | | |  |  | |  |  | | | | | | |
|  | <R500 | 1 | | |  |  | |  |  | | | | | | |
|  | R500-R1499 | 2 | | |  |  | |  |  | | | | | | |
|  | R1500-R2999 | 3 | | |  |  | |  |  | | | | | | |
|  | >R3000 | 4 | | |  |  | |  |  | | | | | | |
| ***B) I am going to ask you questions on your lifestyle and diabetes management***  ***(Please choose the best answer in the following statements)*** | | | | | |  |  |  | | | |  | |  | |
| **1) What is your cigarette smoking status?** |  | | | | |  |  | | | | |  | |  | |
| **If “Never Smoked” go to question 3** | Currently smoking | 1 | | |  |  | | | |  | | |  | |  |
|  | Previously smoking | 2 | | |  |  |  |  |  |  | | |  | |  |
|  | Never Smoked | 3 | | |  |  |  |  |  |  | | |  | |  |
| **2) Has your doctor, nurse or other health practitioner counselled you to about stop smoking?** | | | | | |  |  | | | | |  | |  | |
|  | Yes | 1 | | |  |  | | | |  | | |  | |  |
|  | No | 2 | | |  |  |  |  |  |  | | |  | |  |
| **3) Over the past 7 days, how often did you take a walk, walking a dog, walking to the shop etc.?** | | | | | |  | | | |  | | |  | |  |
|  | Never (0 days) | 1 | | |  |  | | | |  | | |  | |  |
|  | Seldom (1 or 2 days) | 2 | | |  |  |  |  |  |  | | |  | |  |
|  | Sometimes (3 or 4 days) | 3 | | |  |  |  |  |  |  | | |  | |  |
|  | Often (5-7 days) | 4 | | |  |  |  |  |  |  | | |  | |  |
| **4) Have you received advice / information about physical activities/exercise and diabetes from a doctor and nurse in the past?** | | | | | |  |  | | | | |  | |  | |
|  | Yes | | 1 | |  |  | | | |  | | |  | |  |
|  | No | | 2 | |  |  |  |  |  |  | | |  | |  |
| **5) Are you following a diabetic eating plan** | | | | | |  |  | | | | |  | |  | |
|  | Yes | | | 1 |  |  | | | |  | | |  | |  |
|  | No | | | 2 |  |  | | | |  | | |  | |  |

| **6) Have you received advice/information about what you eat as someone with diabetes from a doctor, dietician or a nurse in the past?** |  |  |  | |  |
| --- | --- | --- | --- | --- | --- |
|  | Yes | 1 |  |  |  |
|  | No | 2 |  |  |  |
| **7) Does your blood glucose ever get tested?** | | | | |  |
|  | Yes | 1 |  | |  |
|  | No | 2 |  |  |  |
| **8) If Yes, how often** | | | |  | |
|  | Once a day | 1 |  |  | |
|  | 3 or 4 times a day | 2 |  | |  |
|  | 1 or 2 times a week | 3 |  |  |  |
|  | Occasionally as needed | 4 |  |  |  |
| **9) When last was your urine tested** | | | | |  |
|  | Today | 1 |  | |  |
|  | One Month ago | 2 |  |  |  |
|  | 3 months ago | 3 |  |  |  |
|  | 6 months ago | 4 |  |  |  |
|  | One Year ago | 5 |  |  |  |
|  | Not done at all | 6 |  |  |  |
| **10) When last was your blood pressure checked?** |  |  |  | |  |
|  | Today | 1 |  | |  |
|  | One Month ago | 2 |  |  |  |
|  | 3 months ago | 3 |  |  |  |
|  | 6 months ago | 4 |  |  |  |
|  | One Year ago | 5 |  |  |  |
|  | Not done at all | 6 |  |  |  |
| **11) When last was your cholesterol (fat in your blood) checked?** | | |  |  |  |
|  | Today | 1 |  | |  |
|  | One Month ago | 2 |  |  |  |
|  | 3 months ago | 3 |  |  |  |
|  | 6 months ago | 4 |  |  |  |
|  | One Year ago | 5 |  |  |  |
|  | Not done at all | 6 |  |  |  |
| **12) When last did a doctor/nurse look at the back of your eye or took a photo of the back of your eye?** | | | | |  |
|  | Today | 1 |  | |  |
|  | One Month ago | 2 |  |  |  |
|  | 3 months ago | 3 |  |  |  |
|  | 6 months ago | 4 |  |  |  |
|  | One Year ago | 5 |  |  |  |
|  | Not done at all | 6 |  |  |  |

| **13) When last did you have your feet examine?** | | | |
| --- | --- | --- | --- |
|  | Today | 1 |  |
|  | One Month ago | 2 |  |
|  | 3 months ago | 3 |  |
|  | 6 months ago | 4 |  |
|  | One Year ago | 5 |  |
|  | Not done at all | 6 |  |
| **14) When was your last visit to your doctor** | | | |
|  | Today | 1 |  |
|  | One Month ago | 2 |  |
|  | 3 months ago | 3 |  |
|  | 6 months ago | 4 |  |
|  | One Year ago | 5 |  |
|  | Not done at all | 6 |  |

| ***C) I am now going to ask you about your Diabetes*** | | | |
| --- | --- | --- | --- |
| 1. **How many years have you lived with diabetes?** | | | |
|  | Less than 5 Years | 1 |  |
|  | 5-10 Years | 2 |  |
|  | 11-16 Years | 3 |  |
|  | >17 years | 4 |  |
| 1. **Have you ever received advice on how to live with diabetes from a doctor, dietitian or nurse in the past?** | | | |
|  | Yes | 1 |  |
|  | No | 2 |  |
| 1. **Do you take medication for diabetes that was prescribed by a doctor or a nurse** | | | |
|  | Yes | 1 |  |
|  | No | 2 |  |
| **3a ) If yes, then please indicate the type of treatment** | | | |
|  | Insulin Injections | 1 |  |
|  | Pills | 2 |  |
|  | Both | 3 |  |
| **4) If you are using insulin, do you need any help administering the injection?** | | | |
|  | Yes | 1 |  |
|  | No | 2 |  |
| **4a) If Yes what kind of help do you need?** | | | |
|  | **Drawing up the solution** | **1** |  |
|  | **Injecting** | **2** |  |
|  | **Other** | **3** |  |
| **5) Have you ever stopped taking your medication without telling your doctor, because you felt unwell when you took it?** | | | |
|  | Regularly | 1 |  |
|  | Sometimes | 2 |  |
|  | Rarely | 3 |  |
|  | No | 4 |  |
| **6) When you feel like your diabetes is under control, do you stop taking your medicine?** | | | |
|  | **Yes** | **1** |  |
|  | **No** | **2** |  |
|  | **Sometimes** | **3** |  |
| 1. **Many people find taking their medicine every day is difficult. Do you ever find it difficult taking your medication every day?** | | | |
|  | **Yes** | **1** |  |
|  | **No** | **2** |  |
|  | **Not Sure** | **3** |  |
| **7a Do you find it difficult sticking to your diabetes treatment plan** | | | |
|  | **Yes** | **1** |  |
|  | **No** | **2** |  |
|  | **Not sure** | **3** |  |

| 1. **Do YOU ever test your blood glucose?** | | | |
| --- | --- | --- | --- |
|  | **Yes** | **1** |  |
|  | **No** | **2** |  |
| **8a If Yes, How often** | | | |
|  | **Once a day** | **1** |  |
|  | **3-4 times a day** | **2** |  |
|  | **1 or 2 times a week** | **3** |  |
|  | **Occasionally** | **4** |  |
| **8 b) Who does this** | | | |
|  | **Myself** | **1** |  |
|  | **Family Member** | **2** |  |
|  | **Health Care workers** | **3** |  |
|  | **Other** | **4** |  |
| 1. **Are You receiving medication for any other chronic diseases?** | | | |
|  | **Hypertension** | **1** |  |
|  | **Heart Disease** | **2** |  |
|  | **Traditional Medication** | **3** |  |
|  | **Other** | **4** |  |
|  | **Don’t know** | **5** |  |
| 1. **Which if any, of the following problems sometimes associated with diabetes have you experience?** | | | |
|  | **Low Blood sugar (<4mmol)** | **1** |  |
|  | **High Blood sugar (>10 mmol)** | **2** |  |
|  | **Heart problems** | **3** |  |
|  | **Sexual difficulties** | **4** |  |
|  | **Damage to the back of the eye** | **5** |  |
|  | **Nerve damage (e.g. numbness or tingling of the hands or feet, or foot ulcers)** | **6** |  |
|  | **Kidney problems** | **7** |  |
|  | **None of the above.** | **8** |  |
|  | **Don’t know** | **9** |  |
| 1. **Have you been hospitalized for diabetes in the past 12 months?** | | | |
|  | **Yes** | **1** |  |
|  | **No** | **2** |  |
|  |  |  |  |
|  |  |  |  |

| ***D) I am going to ask you questions concerning diabetes.***  ***(Please choose the appropriate answers(s) to the following statements)*** | | | |
| --- | --- | --- | --- |
| 1. **Diabetes is a condition in which the body contains** | | | |
|  | **A higher level of sugar in blood than normal** | **1** |  |
|  | **A lower level of sugar in the blood than normal** | **2** |  |
|  | **I don’t know** | **3** |  |
| 1. **The major cause of diabetes is:** | | | |
|  | **Too much insulin in the body** | **1** |  |
|  | **Too little insulin in the body** | **2** |  |
|  | **I don’t know** | **3** |  |
|  |  |  |  |
| 1. **The symptom(s) of diabetes is/are:** | | | |
|  | **Increased number of times of urination** | **1** |  |
|  | **Increased thirst and hunger** | **2** |  |
|  | **Increased tiredness** | **3** |  |
|  | **Slow healing of wounds** | **4** |  |
|  | **I don’t know** | **5** |  |
| 1. **Diabetes, if not treated** | | | |
|  | **Can lead to eye problems** | **1** |  |
|  | **Can lead to kidney problems** | **2** |  |
|  | **Can lead to foot ulcers** | **3** |  |
|  | **Can lead to heart problems** | **4** |  |
|  | **I don’t know** | **5** |  |
| 1. **The most accurate method of monitoring diabetes is:** | | | |
|  | **Checking blood sugar levels** | **1** |  |
|  | **Checking sugar in the urine** | **2** |  |
|  | **HbA1c** | **3** |  |
|  | **I don’t know** | **4** |  |
| 1. **In a diabetic patient, high blood pressure can increase or worsen:** | | | |
|  | **The risk of heart attack** | **1** |  |
|  | **The risk of stroke** | **2** |  |
|  | **The risk of eye problems** | **3** |  |
|  | **The risk of kidney problems** | **4** |  |
|  | **I don’t know** | **5** |  |
| 1. **To live healthily when you have diabetes, the lifestyle changes required are:** | | | |
|  | **Weight reduction** | **1** |  |
|  | **To Stop smoking** | **2** |  |
|  | **To Stop alcohol intake** | **3** |  |
|  | **Healthy diet** | **4** |  |
|  | **Regular physical exercise** | **5** |  |
|  | **All of the above** | **6** |  |
|  | **I don’t know** | **7** |  |
|  |  |  |  |
| 1. **A diabetic patient should have his or her eyes checked:** | | | |
|  | **Once a year** | 1 |  |
|  | Once every six months | 2 |  |
|  | Need not check at all | 3 |  |
| 1. **A well-balanced diet includes:** | | | |
|  | **Green leafy vegetables** | 1 |  |
|  | **High fibre foods** | 2 |  |
|  | **Low sugar, oil and fat** | 3 |  |
|  | **All the mentioned** | 4 |  |
|  | I don’t know | 5 |  |
| 1. **For proper foot care, a diabetic patient:** | | | |
|  | **Should inspect and wash the feet daily** | 1 |  |
|  | **Should select the best possible footwear** | 2 |  |
|  | Should walk barefoot inside and outside the house | 3 |  |
|  | **Should not walk barefoot inside and outside the house** | 4 |  |
| 1. **Upon control of diabetes, the medicines:** | | | |
|  | Can’t be stopped immediately | 1 |  |
|  | Can be stopped after one month | 2 |  |
|  | **Should be continued for life** | 3 |  |
|  | I don’t know | 4 |  |
| 1. **Do you have any cultural or religious practices or beliefs that influence how you care for your diabetes?** | | | |
|  | Yes | 1 |  |
|  | No | 2 |  |
|  | If Yes please describe:……………………………………………………………………………………………………………………………………………………………………………………………………………………………………………………………………………………………………………………………………………………………………………… |  |  |
| 1. **It is helpful for us to understand health needs in, different communities for this reason,**   **we would like to ask you which of the following groups You put yourself in?** | | | |
|  | **Black African** | 1 |  |
|  | **White African** | 2 |  |
|  | **Coloured African** | 3 |  |
|  | **Indian (Asian)** | 4 |  |
|  | **Other (Specify)** | 5 |  |

**E. Social Support Scale**

**You may think that some of the next sets of questions have already been asked but here we are asking about your family and friends**

**Q1. My family or friends help and support me a lot to: (*Please Circle one answer for each line*)**

|  | **Strongly Disagree** | **Somewhat Disagree** | **Neutral** | **Somewhat Agree** | **Strongly Agree** | **Does Not Apply** |
| --- | --- | --- | --- | --- | --- | --- |
| **a. Follow my meal plan.** | 1 | 2 | 3 | 4 | 5 | N/A |
| **b. Take my medicine.** | 1 | 2 | 3 | 4 | 5 | N/A |
| **c. Take care of my feet.** | 1 | 2 | 3 | 4 | 5 | N/A |
| **d. Get enough physical activity.** | 1 | 2 | 3 | 4 | 5 | N/A |
| **e. Test my sugar.** | 1 | 2 | 3 | 4 | 5 | N/A |
| **f. Handle my feelings about diabetes.** | 1 | 2 | 3 | 4 | 5 | N/A |

**Q2. My family or friends: (*Please Circle one answer for each line*)**

|  | **Strongly Disagree** | **Somewhat Disagree** | **Neutral** | **Somewhat Agree** | **Strongly Agree** | **Does Not Apply** |
| --- | --- | --- | --- | --- | --- | --- |
| **a. Accept me and my diabetes.** | 1 | 2 | 3 | 4 | 5 | N/A |
| **b. Feel uncomfortable about me because of my diabetes.** | 1 | 2 | 3 | 4 | 5 | N/A |
| **c. Encourage or reassure me about my diabetes.** | 1 | 2 | 3 | 4 | 5 | N/A |
| **d. Discourage or upset me about my diabetes.** | 1 | 2 | 3 | 4 | 5 | N/A |
| **e. Listen to me when I want to talk about my diabetes.** | 1 | 2 | 3 | 4 | 5 | N/A |
| **f. Nag me about diabetes.** | 1 | 2 | 3 | 4 | 5 | N/A |

***F. (Please Note this section should be completed by Field Workers)***

Glycaemic + BP data in the last three visits (from the patient’s record)

| **Item** | **Date** | **Visit I** | **Visit II** | **Visit III** |
| --- | --- | --- | --- | --- |
| ***Fasting blood glucose***  ***Level/mg/dl*** |  |  |  |  |
| ***HbA1c %*** |  |  |  |  |
| ***Blood Pressure*** |  |  |  |  |
