# Supplementary material for: Does social support effect knowledge and diabetes self-management practices in older persons with Type 2 diabetes attending primary care clinics in Cape Town, South Africa?
Source: PLoS One. 2020 Mar 13;15(3):e0230173. doi: 10.1371/journal.pone.0230173 (PMC7069645; doi:10.1371/journal.pone.0230173)
Supplement: S1 Table — (DOCX) [file pone.0230173.s002.docx]

**Table. S1. Mean Diabetes Knowledge, self-management practice and social support scores of the participants by socio-demographic characteristics.**

| Variables | | N | Total knowledge score | | Total self-management practice score | | Total social support score | |
| --- | --- | --- | --- | --- | --- | --- | --- | --- |
|  |  |  | **Mean** | **(SD)** | **Mean** | **(SD)** | **Mean** | **(SD)** |
| Sex | **Male** | 126 | 45.0 | 12.7 | 54.2 | 19.9 | 79.7 | 20.7 |
|  | **Female** | 280 | 47.7 | 13.1 | 53.6 | 17.3 | 75.3 | 20.8 |
|  | *** P value** |  | 0.053 |  | 0.76 |  | 0.048* |  |
| Age group | **60-69** | 257 | 47.8 | 11.9 | 53.3 | 18.7 | 76.7 | 19.9 |
|  | **70-79** | 121 | 45.6 | 15.2 | 54.3 | 16.9 | 76.4 | 22.7 |
|  | **80 or above** | 28 | 42.6 | 11.3 | 55.2 | 18.5 | 79.3 | 20.8 |
|  | **^+^ P value** |  | 0.21 |  | 0.10 |  | 0.54 |  |
| Level of Education |  |  |  |  |  |  |  |  |
|  | **None / some primary school** | 233 | 45.7 | 12.4 | 51.5 | 18.2 | 77.7 | 20.4 |
|  | **Some high school** | 150 | 47.6 | 13.5 | 54.9 | 17.5 | 75.1 | 21.0 |
|  | **Matric / Tertiary** | 12 | 50.1 | 10.1 | 58.4 | 17.7 | 77.8 | 20.3 |
|  | **^#^ P value** |  | 0.008**^#^** |  | 0.180 |  | 0.817 |  |
| Marital status | **Single** | 44 | 41.2 | 12.9 | 54.5 | 17.4 | 78.7 | 22.7 |
|  | **Married** | 209 | 46.7 | 12.5 | 54.4 | 18.5 | 77.4 | 20.1 |
|  | **Divorced/ Separated** | 65 | 45.5 | 15.1 | 50.1 | 18.00 | 74.5 | 20.3 |
|  | **≠Others** | 88 | 49.4 | 12.7 | 54.2 | 17.3 | 75.2 | 21.8 |
|  | **^+^ P value** |  | 0.008**^+^** |  | 0.68 |  | 0.83 |  |
| Race |  |  |  |  |  |  |  |  |
|  | **Coloured (mixed race)** | 312 | 47.0 | 12.7 | 53.2 | 18.3 | 76.5 | 20.7 |
|  | **Other (White, Black, Indian and other** | 94 | 47.2 | 13.3 | 55.9 | 17.6 | 77.0 | 21.7 |
|  | *** P value** |  | 0.21 |  | 0.20 |  | 0.84 |  |
| Who are Living with |  |  |  |  |  |  |  |  |
|  | **Spouse** | 52 | 49.6 | 12.1 | 55.4 | 18.3 | 67.7 | 21.0 |
|  | **Family member** | 246 | 46.8 | 13.1 | 54.3 | 17.8 | 77.3 | 20.6 |
|  | **Friend/** **More than one** | 83 | 45.7 | 14.7 | 55.5 | 19.5 | 82.3 | 18.0 |
|  | **Alone** | 25 | 45.0 | 12.3 | 50.6 | 18.5 | 75.0 | 21.5 |
|  | **^+^ P value** |  | 0.24 |  | 0.35 |  | 0.001**^+^** |  |
| Monthly family income |  |  |  |  |  |  |  |  |
|  | **Less than R 1 500** | 350 | 46.6 | 12.9 | 54.1 | 18.4 | 77.0 | 21.0 |
|  | **R 1 500 or more** | 46 | 47.6 | 14.3 | 53.1 | 14.0 | 74.8 | 20.1 |
|  | *** P value** |  | 0.63 |  | 0.69 |  | 0.48 |  |
| * t-test for Equality of Means (Sig. (2-tailed), ^+^ ONE-WAY ANOVA test,  ≠ Others: have a joint household, live together. cohabit | | | | | | | | |
