# Supplementary material for: Does social support effect knowledge and diabetes self-management practices in older persons with Type 2 diabetes attending primary care clinics in Cape Town, South Africa?
Source: PLoS One. 2020 Mar 13;15(3):e0230173. doi: 10.1371/journal.pone.0230173 (PMC7069645; doi:10.1371/journal.pone.0230173)
Supplement: S2 Table — (DOCX) [file pone.0230173.s003.docx]

**Table S2. Mean Diabetes Knowledge, self-management practice and social support scores by clinical characteristics of the Participants.**

| Variables |  | N | Total knowledge score | | Total self-management practice score | | Total social support score | |
| --- | --- | --- | --- | --- | --- | --- | --- | --- |
|  |  |  | **Mean** | **(SD)** | **Mean** | **(SD)** | **Mean** | **(SD)** |
| Duration of Diabetes |  |  |  |  |  |  |  |  |
|  | Less than 5 years | 125 | 45.3 | 14.03 | 55. | 16.8 | 77.0 | 19.8 |
|  | 5-10 years | 158 | 47.3 | 13.07 | 52.0 | 19.5 | 75.2 | 22.3 |
|  | 11-16 years | 84 | 48.2 | 11.8 | 53.7 | 19.0 | 79.0 | 18.9 |
|  | >17 years | 36 | 46.9 | 12.1 | 56.0 | 13.8 | 76.3 | 22.5 |
|  | **^+^P value** |  | 0.42 |  | 0.34 |  | 0.59 |  |
| Type of medication used |  |  |  |  |  |  |  |  |
|  | Insulin injections | 28 | 43.3 | 13.9 | 52.9 | 17.1 | 77.9 | 26.0 |
|  | Pills | 250 | 47.6 | 13.1 | 52.5 | 17.9 | 76.7 | 20.1 |
|  | Both | 125 | 46.1 | 12.6 | 56.2 | 18.7 | 76.8 | 21.2 |
|  | **+ P value** |  | 0.19 |  | 0.18 |  | 0.95 |  |
| Have you experienced low blood sugar | No | 333 | 45.9 | 13.2 | 53.2 | 18.4 | 77.02 | 21.5 |
|  | Yes | 73 | 51.2 | 11.2 | 56.7 | 16.5 | 75.2 | 18.0 |
|  | *** P value** |  | .001 |  | 0.12 |  | .45 |  |
| Have you experienced high blood sugar | No | 211 | 45.1 | 13.5 | 55.0 | 16.6 | 78.5 | 19.8 |
|  | Yes | 195 | 48.7 | 12.2 | 52.2 | 19.6 | 74.7 | 21.8 |
|  | *** P value** |  | .005***** |  | .092 |  | .064 |  |
| Receiving medication for chronic hypertension |  |  |  |  |  |  |  |  |
|  | No | 100 | 45.2 | 13.0 | 54.18 | 17.8 | 75.8 | 21.27 |
|  | Yes | 306 | 47.4 | 13.0 | 53.7 | 18.3 | 76.9 | 20.8 |
|  | *** P value** |  | 0.15 |  | 0.83 |  | .63 |  |
| Receiving medication for heart disease |  |  |  |  |  |  |  |  |
|  | **No** | 345 | 46.4 | 13.0 | 54.7 | 17.9 | 76.6 | 20.4 |
|  | **Yes** | 61 | 49.4 | 13.0 | 48.7 | 18.8 | 77.0 | 23.7 |
|  | **^*^ P value** |  | .097 |  | .017**^*^** |  | 0.88 |  |
| Receiving medication for other chronic disease(s) | **No** | 268 | 45.9 | 13.4 | 53.7 | 18.6 | 76.2 | 21.0 |
|  | **Yes** | 138 | 48.7 | 11.9 | 53.9 | 17.1 | 77.7 | 20.7 |
|  | **^*^ P value** |  | 0.038 |  | 0.93 |  | 0.48 |  |
