# Supplementary material for: Does social support effect knowledge and diabetes self-management practices in older persons with Type 2 diabetes attending primary care clinics in Cape Town, South Africa?
Source: PLoS One. 2020 Mar 13;15(3):e0230173. doi: 10.1371/journal.pone.0230173 (PMC7069645; doi:10.1371/journal.pone.0230173)
Supplement: S3 Table — (DOCX) [file pone.0230173.s004.docx]

**Table S3. Association of socio-demographic variables, HbA1c and social support with knowledge score**

| **Variable** | ***β*** | **95% CI** | **P value** |
| --- | --- | --- | --- |
| **Female** | 0.660 | -0.285; 1.604 | 0.160 |
| **Age group, years**  **(ref = 55-69 years)** | | | |
| 70-79 years | -0.461 | -1.495; 0.572 | 0.380 |
| >80 years | -1.893 | -3.754; -0.031 | **0.046** |
| **Education level**  **(ref = None / some primary school)** | | | |
| Some or completed High school/Tertiary education | 0.639 | -0.273; 1.550 | 0.169 |
| I**ncome** >R1500  (ref = <R1500) | 0.324 | -1.013; 1.661 | 0.634 |
| **Living alone**  (ref = Living with Family/friends/spouse) | 0.111 | -1.668; 1.889 | 0.903 |
| **HbA1c >8%**  (ref = HbA1c <8%) | -0.272 | -1.183; 0.638 | 0.557 |
| Social support | -0.018 | -0.093; 0.058 | 0.649 |

**Multivariable linear regression was used to evaluate the associations between knowledge (dependant variable) and sociodemographic variables (gender, age group, education level, income, living environment, glycaemic control and social support (independent variables).**

**Adjusted R-squared – 1.0%, Prob > F = 0.206**
