# Supplementary material for: Does social support effect knowledge and diabetes self-management practices in older persons with Type 2 diabetes attending primary care clinics in Cape Town, South Africa?
Source: PLoS One. 2020 Mar 13;15(3):e0230173. doi: 10.1371/journal.pone.0230173 (PMC7069645; doi:10.1371/journal.pone.0230173)
Supplement: S4 Table — (DOCX) [file pone.0230173.s005.docx]

**Table S4. Association of socio-demographic variables, HbA1c and social support with self-management practice score**

| **Variable** | ***β*** | **95% CI** | **P value** |
| --- | --- | --- | --- |
| Female | 1.230 | -0.372; 2.831 | 0.131 |
| Age group, years  (ref = 55-69 years) | | | |
| 70-79 years | 0.510 | -1.178; 2.199 | 0.551 |
| >80 years | 0.443 | -2.505; 3.392 | 0.767 |
| Education level  (ref = None / some primary school) | | | |
| Some or completed High school/Tertiary education | -0.038 | -1.592; 1.516 | 0.961 |
| Income >R1500  (ref = <R1500) | 3.434 | 0.797; 6.070 | 0.011 |
| Living alone  (ref = Living with Family/friends/spouse) | -0.725 | -3.496; 2.047 | 0.606 |
| HbA1c >8%  (ref = HbA1c <8%) | 0.117 | -1.427; 1.660 | 0.881 |
| Social support | -0.061 | -0.065; 0.188 | 0.340 |

**Multivariable linear regression was used to evaluate the associations between self-management practice score (dependant variable) and sociodemographic variables (gender, age group, education level, income, living arrangements), glycaemic control and social support (independent variables).**

**Adjusted R-squared – 1.3%, Prob > F = 0.275**
